# Epididymal disjunction anomalies in undescended testis – a factor associated with spermatic obstruction

**DOI:** 10.1590/S1677-5538.IBJU.2022.99.07

**Published:** 2022-01-10

**Authors:** Natasha T. Logsdon, Carla M. Gallo, Francisco J. B. Sampaio, Luciano A. Favorito

**Affiliations:** 1 Universidade do Estado do Rio de Janeiro – UERJ Unidade de Pesquisa Urogenital Rio de Janeiro RJ Brazil Unidade de Pesquisa Urogenital, Universidade do Estado do Rio de Janeiro – UERJ, Rio de Janeiro, RJ

**Keywords:** Cryptorchidism, Epididymis, Infertility

## Abstract

**Background:**

To analyze the incidence of epididymal anomalies (EAs) associated to spermatic obstruction in patients with undescended testis (UT) according to testicular position and age.

**Materials and Methods:**

We studied 87 patients (110 testis) with cryptorchidism and analyzed the presence of EAs correlated with the testicular position, age and patency of the processus vaginalis (PV). To analyze the relations between the testis and epididymis we considered three situations: (a) Normal pattern: the epididymis was attached to the testis at the head and tail and epididymis totally attached to the testis; (b) EAs: when the epididymis was attached to the testis only at the head (Figure-1A) and (c) EAs associated to spermatic obstruction: epididymis was attached to the testis only at the tail (Figure-1B) and when there are no visible connection between testis and epididymis (Figure-1C). We used the Wilcoxon-Mann-Whitney test and the Chi-square test for contingency analysis (p <0.05).

**Results:**

The mean age of the patients was 5.18 years (SD=2.867). Of 110 testes analyzed, 14 were abdominal (12.72%); 83 inguinal (75.45%) and 13 suprascrotal (11.81%). Normal relationships between testis and epididymis were observed in 54 patients (62.1%) with no significant differences in relation to the patient's age (p=0.666). Epididymal tail disjunction was observed in 23 patients (26.44%), with no significant differences in relation to age (p=0.59). EAs associated to spermatic obstruction were observed in 16 patients (18.4%), also with no significant differences in relation to age (p=0.684). We did not observe significant correlation between the testis position and the incidence of EAs (p=0.119). We did not observe significant correlations between patency of the PV (64.7%) and incidence of EAs (p=0.742).

**Conclusions:**

Epididymal anomalies associated with spermatic obstruction are present in almost 20% of undescended testes, without significant correlation with age, testicular position and patency of the PV. This information needs to be correlated to the infertility risk of this congenital anomaly.

## INTRODUCTION

Undescended testis can be associated with several anatomical anomalies. Epididymal anomalies (EAs) and patency of the processus vaginalis (PV) are among the most frequent, with a highly variable incidence reported in the literature: from 36 to 79% (1-4). Undescended testis is associated with greater chance of testicular cancer and infertility (5, 6). Infertility in patients with undescended testis may result from obstruction associated with epididymal disjunction anomalies and epididymis atresia (7, 8).

Several studies have examined the relationship of EAs and patency of the PV (9-11). On the other hand, analysis of the correlation between the incidence of anomalies associated with spermatic obstruction in undescended testis (head disjunction and total epididymal disjunction) with testis position, patient age and patency of the PV are rare in literature.

We hypothesized that disjunction of epididymal head and total disjunction of epididymis are usual in undescended testes and more frequent in testes situated in high position and with patency of the PV. The aim of this article is to evaluate the incidence of epididymal anomalies associated with spermatic obstruction in patients with undescended testis (UT) according to testicular position, patient age and patency of the PV.

## MATERIALS AND METHODS

This retrospective single-center study was approved and was carried out in accordance with the ethical standards of the hospital's institutional committee on human experimentation - IRB number 30706720.8.0000.5259.

From January 2011 to January 2020, we studied 87 patients with cryptorchidism (110 testes). All patients underwent orchidopexy and the testes were divided into three groups according to their position: A) abdominal: testes located above the inner inguinal ring; B) inguinal: testes located between the inner and outer inguinal rings; and C) suprascrotal: testes located below the outer inguinal ring. During surgery, three parameters were analyzed: A) testicular position; B) relationship between testis and epididymis; and C) persistence of processus vaginalis (PV).

To analyze the relations between the testis and epididymis, we used a previous classification (12, 13): Type I - epididymis attached to the testis at the head and tail; Type II - epididymis totally attached to the testis; Type III - epididymis attached to the testis only at the head; Type IV - epididymis attached to the testis only at the tail; Type V - no visible connection between testis and epididymis; and Type VI - epididymal atresia. Type I and II relationships are considered normal while the other types are considered epididymal anomalies (EAs) (12).

**Figure 1:**
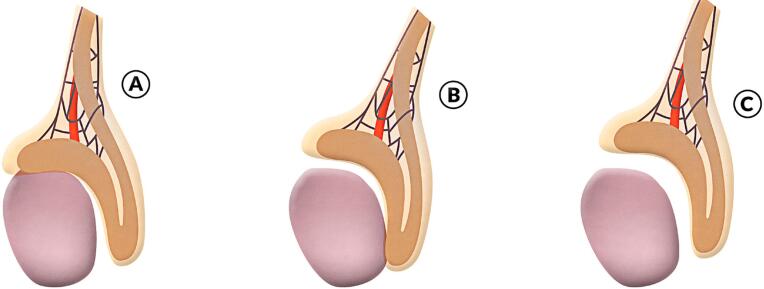
Schematic drawing showing the epididymal disjunction anomalies: A) disjunction between testis and epididymal tail; B) disjunction between testis and epididymal head; and C) total disjunction between testis and epididymis.

Figure-1 depicts the disjunction anomalies of the epididymis. The EAs with head disjunction and total disjunction (Type IV and V) were analyzed together because they can lead to infertility by the presence of a spermatic obstruction preventing the passage of sperm towards the epididymis, especially if they are present bilaterally in cases of undescended testis. To analyze the PV, we considered two situations: (a) complete obliteration of the PV between the internal inguinal ring and the upper pole of the testis; and (b) complete patency of the PV.

### Statistical Analysis

All parameters were statistically processed and graphically described. The Wilcoxon-Mann-Whitney test was used for comparison of quantitative data (p <0.05). The Chi-square test was used to analyze the population contingency under study (p<0.05), calculated by Graph Pad.

## RESULTS

All the 87 patients were aged between 1 and 10 years (mean=5.18/SD=2.867). Of these patients, 29 (33.33%) had cryptorchidism on the right side, 35 (40.22%) on the left side and 23 (26.43%) were bilateral. Of 110 testes, 14 were abdominal (12.72%), 83 inguinal (75.45%) and 13 suprascrotal (11.81%). The correlation between EAs and patient age can be seen in Table-1. The relationships between testes position, epididymal anomalies and patency of the PV are reported in Table-2. In Table-3 we can observe all the data of the 87 studied patients.

**Table 1:** The table shows the correlation between epidididymal anomalies with age in patients with undescended testis. The relationships between testis and epididymis was classified as Type I when epididymis attached to the testis at the head and tail; Type II - epididymis totally attached to the testis; Type III.

Relationships Between Testis and Epididymis		Age (years)					
		1 to 2 years	3 to 5 years	6 to 10 years	Total	Mean	SD
		N(%)	N(%)	N(%)			
Normal Relationship (Types I and II)	No	5(29.4)	12(42.9)	16(38.1)	33	5,48	2,917
	Yes	12(70.6)	16(57.1)	26(61.9)	54	5,00	2,848
	Total	17(100)	28(100)	42(100)	87	5,18	2,867
Epididymal Tail Disjunction (Type III)	No	13(76.5)	19(67.9)	33(78.6)	65	5,22	2,923
	Yes	4(23.5)	9(32.1)	9(21.4)	22	5,09	2,759
	Total	17(100)	28(100)	42(100)	87	5,18	2,867
Epididymal Disjunctions associated to spermatic obstruction (Types IV and V)	No	15(88.2)	23(82.1)	33(78.6)	71	5,00	2,788
	Yes	2(11.8)	5(17.9)	9(21.4)	16	6,00	3,162
	Total	17(100)	28(100)	42(100)	87	5,18	2,867

**Table 2:** The table shows the correlation between epidididymal anomalies with testicular position and patency of processus vaginalis in 87 patients studied. The relationships between testis and epididymis was classified as Type I when epididymis attached to the testis at the head and tail; Type II - epididymis totally attached to the testis; Type III - epididymis attached to the testis only at the head; Type IV - epididymis attached to the testis only at the tail; Type V – no visible connection between the testis and epididymis.

RELATIONSHIPS BETWEEN TESTIS AND EPIDIDIMYS	TESTICULAR POSITION		PROCESSUS VAGINALIS		
	**Abdominal**	**Canal**	**Supra-Escrotal**	**Open**	**Close**
	n (%)	n (%)	n (%)	n (%)	n (%)
Normal (Types I and II)	7 (50)	50 (60.2)	12 (92.3)	45 (60.8)	24 (66.7)
**Tail disjunction (Type III)**	5 (35.7)	18 (21.7)	0	17 (23)	6 (16.7)
**Anomalies Associated to Infertility (Types IV and V)**	2 (14.3)	15 (18.1)	1 (7.7%)	12 (16.2)	6 (16.7)
**Total**	**14 (100)**	**83 (100)**	**13 (100)**	**74 (67.27)**	**36 (32.72)**

**Table 3:** The table shows the data in 87 studied patients with cryptorchidism in order of age. Type I - epididymis attached to the testis at the head and tail; Type II - epididymis totally attached to the testis; Type III - epididymis attached to the testis only at the head; Type IV - epididymis attached to the testis only at the tail; Type V – no visible connection between testis and epididymis; and Type VI – epididymal atresia.

PATIENTS	AGE	SIDE	POSITION	EPIDIDYMIS	PROCESSUS VAGINALIS
1	1 year	Right	Inguinal	Type I	Patent
1	1 year	Left	Inguinal	Type I	Patent
2	1 year	Left	Inguinal	Type V	Patent
3	1 year	Right	Abdominal	Type III	Patent
4	1 year	Right	Abdominal	Type III	Patent
5	1 year	Right	Abdominal	Type II	Patent
6	1 year	Left	Inguinal	Type V	Patent
7	1 year	Right	Inguinal	Type I	Patent
7	1 year	Left	Inguinal	Type I	Patent
8	1 year	Left	Inguinal	Type I	Patent
9	1 year	Left	Inguinal	Type I	Patent
10	1 year	Right	Suprascrotal	Type I	Patent
11	1 year	Right	Suprascrotal	Type I	Patent
12	2 years	Right	Inguinal	Type I	Patent
12	2 years	Left	Inguinal	Type III	Patent
13	2 years	Right	Inguinal	Type II	Obliterated
PATIENTS	AGE	SIDE	POSITION	EPIDIDYMIS	PROCESSUS VAGINALIS
14	2 years	Left	Inguinal	Type III	Patent
15	2 years	Right	Inguinal	Type I	Patent
16	2 years	Right	Inguinal	Type II	Obliterated
17	2 years	Right	Suprascrotal	Type I	Obliterated
17	2 years	Left	Suprascrotal	Type II	Obliterated
18	3 years	Right	Suprascrotal	Type II	Obliterated
19	3 years	Left	Inguinal	Type I	Patent
20	3 years	Left	Abdominal	Type III	Patent
21	3 years	Right	Inguinal	Type IV	Obliterated
21	3 years	Left	Inguinal	Type I	Patent
22	3 years	Left	Abdominal	Type II	Patent
23	3 years	Left	Abdominal	Type II	Patent
24	3 years	Right	Inguinal	Type I	Patent
25	3 years	Right	Abdominal	Type III	Patent
26	3 years	Right	Inguinal	Type I	Patent
27	3 years	Left	Inguinal	Type I	Obliterated
28	3 years	Right	Abdominal	Type III	Patent
29	3 years	Right	Inguinal	Type I	Patent
PATIENTS	AGE	SIDE	POSITION	EPIDIDYMIS	PROCESSUS VAGINALIS
30	3 years	Right	Inguinal	Type I	Patent
30	3 years	Left	Inguinal	Type I	Obliterated
31	3 years	Right	Inguinal	Type III	Patent
32	4 years	Right	Inguinal	Type I	Patent
33	4 years	Left	Inguinal	Type III	Patent
34	4 years	Right	Inguinal	Type I	Patent
34	4 years	Left	Inguinal	Type I	Patent
35	4 years	Right	Inguinal	Type I	Patent
35	4 years	Left	Inguinal	Type IV	Patent
36	4 years	Left	Inguinal	Type III	Patent
37	4 years	Right	Inguinal	Type I	Patent
37	4 years	Left	Inguinal	Type I	Patent
38	4 years	Right	Inguinal	Type I	Patent
39	4 years	Left	Inguinal	Type IV	Patent
40	4 years	Left	Inguinal	Type III	Patent
41	4 years	Left	Inguinal	Type III	Patent
42	4 years	Left	Suprascrotal	Type V	Patent
43	5 years	Left	Inguinal	Type III	Patent
44	5 years	Right	Abdominal	Type I	Patent
PATIENTS	AGE	SIDE	POSITION	EPIDIDYMIS	PROCESSUS VAGINALIS
45	5 years	Left	Inguinal	Type II	Patent
46	6 years	Left	Inguinal	Type III	Patent
47	6 years	Right	Inguinal	Type I	Patent
47	6 years	Left	Inguinal	Type I	Patent
48	6 years	Right	Inguinal	Type V	Obliterated
48	6 years	Left	Inguinal	Type V	Patent
49	6 years	Right	Suprascrotal	Type I	Obliterated
49	6 years	Left	Suprascrotal	Type I	Obliterated
50	6 years	Left	Inguinal	Type II	Obliterated
51	6 years	Right	Inguinal	Type II	Obliterated
52	6 years	Left	Abdominal	Type I	Patent
53	6 years	Left	Abdominal	Type I	Patent
54	6 years	Right	Inguinal	Type II	Obliterated
54	6 years	Left	Inguinal	Type I	Patent
55	6 years	Left	Inguinal	Type III	Patent
56	6 years	Right	Inguinal	Type I	Patent
56	6 years	Left	Inguinal	Type I	Patent
57	6 years	Right	Inguinal	Type V	Obliterated
57	6 years	Left	Inguinal	Type V	Patent
PATIENTS	AGE	SIDE	POSITION	EPIDIDYMIS	PROCESSUS VAGINALIS
58	6 years	Right	Inguinal	Type I	Obliterated
58	6 years	Left	Inguinal	Type I	Obliterated
59	6 years	Left	Inguinal	Type I	Obliterated
60	6 years	Right	Suprascrotal	Type I	Obliterated
61	7 years	Right	Inguinal	Type I	Obliterated
57	6 years	Left	Inguinal	Type V	Patent
58	6 years	Right	Inguinal	Type I	Obliterated
58	6 years	Left	Inguinal	Type I	Obliterated
59	6 years	Left	Inguinal	Type I	Obliterated
60	6 years	Right	Suprascrotal	Type I	Obliterated
61	7 years	Right	Inguinal	Type I	Obliterated
62	8 years	Left	Inguinal	Type I	Patent
63	8 years	Right	Inguinal	Type III	Obliterated
63	8 years	Left	Inguinal	Type III	Obliterated
64	8 years	Left	Inguinal	Type I	Obliterated
65	8 years	Left	Inguinal	Type I	Patent
66	8 years	Left	Inguinal	Type I	Obliterated
67	8 years	Right	Inguinal	Type III	Obliterated
68	8 years	Left	Inguinal	Type III	Obliterated
PATIENTS	AGE	SIDE	POSITION	EPIDIDYMIS	PROCESSUS VAGINALIS
69	9 years	Right	Abdominal	Type IV	Patent
70	9 years	Right	Inguinal	Type III	Patent
71	9 years	Left	Inguinal	Type V	Patent
72	9 years	Right	Inguinal	Type I	Obliterated
72	9 years	Left	Inguinal	Type I	Obliterated
73	9 years	Right	Suprascrotal	Type I	Obliterated
73	9 years	Left	Suprascrotal	Type I	Patent
74	9 years	Right	Inguinal	Type II	Patent
74	9 years	Left	Inguinal	Type III	Obliterated
75	9 years	Right	Inguinal	Type IV	Obliterated
76	9 years	Right	Abdominal	Type IV	Patent
77	9 years	Right	Inguinal	Type III	Patent
78	9 years	Left	Inguinal	Type I	Patent
79	9 years	Right	Inguinal	Type III	Obliterated
80	9 years	Right	Inguinal	Type IV	Obliterated
80	9 years	Left	Inguinal	Type I	Obliterated
81	9 years	Right	Inguinal	Type I	Obliterated
82	9 years	Left	Inguinal	Type I	Patent
82	9 years	Right	Inguinal	Type V	Obliterated
PATIENTS	AGE	SIDE	POSITION	EPIDIDYMIS	PROCESSUS VAGINALIS
83	9 years	Left	Inguinal	Type II	Patent
84	9 years	Right	Suprascrotal	Type I	Patent
84	9 years	Left	Suprascrotal	Type I	Patent
85	10 years	Left	Inguinal	Type II	Patent
86	10 years	Left	Abdominal	Type II	Patent
87	10 years	Left	Inguinal	Type IV	Patent

We did not observe epididymal atresia (Type VI anomalies) in our sample. The normal relationships between testis and epididymis (Type I and II) were observed in 54 patients (62.1%), with no significant differences in relation to patient age (p=0.666). We observed normal relationships between testis and epididymis in 12 of the suprascrotal testes (92.3%); 50 of inguinal testes (60.2%) and 7 abdominal testes (50%).

The disjunction of epididymal tail (Type III) was observed in 23 patients (25.3%), with no significant differences in relation to patient age (p=0.59). Disjunction of epididymal tail was observed in 5 (35.7%) of abdominal testes and 18 (21.7%) of inguinal testes. This anomaly was not observed in suprascrotal testes.

Disjunctions of epididymal head and total disjunction of epididymis (anomalies associated with spermatic obstruction - Types IV and V) were observed in 16 patients (18.4%), also with no significant differences in relation to patient age (p=0.684). The anomalies associated with spermatic obstruction were observed in 15 (18.1%) of the inguinal testes, 2 (14.3%) of the abdominal testes and only 1 (7.7%) of the suprascrotal testes.

We did not observe significant correlation between the testis position and the incidence of epididymal anomalies in our sample (p=0.119). We observed 13 patients (11.31%) with bilateral cryptorchidism. In these 13 patients, 8 of them (61.53%) had epididymal anomalies and in 3 of them (23.07%) the epididymal anomalies were bilateral. In bilateral cases of undescended testis the anomalies associated with spermatic obstruction (Types IV and V) were observed in 6 cases (46.15%) and in 2 of them (15.38%) the patients had total disjunction between the testis and epididymis (Type 5) in both sides (Table-3).

Open PV was found in 74 cases (64.7%) of cryptorchidism. We did not observe significant correlations between patency of the PV and incidence of EAs (p=0.742). The normal relationships (Type I and Type II) occurred in 24 (66.7%) of the cases of PV obliterated and in 45 (60.8%) of the cases of PV patency. Epididymal tail disjunction occurred in 6 cases (16.7%) in obliterated PV and 17 cases (23%) in patency of the PV, and the anomalies associated with infertility (Types IV and V) were observed in 6 cases (16.7%) of obliterated PV and 12 cases (16.2%) of patency of the PV.

## DISCUSSION

Epididymal anomalies can occur due to abnormal involution of the mesonephric duct adjacent to the testis (1). The epididymal body and vasal structure arise from the mesonephric tubules and Wolffian duct (14). The union between rete testis and mesonephric tubules begins at around 12 weeks of gestation (14). The epididymal anomalies of disjunction can be explained by the difference in the embryological origin of the epididymis from the mesonephric duct or by changes in vascularization during the development of the mesonephric tubules and their fusion with the testicular tubules (15).

Knowledge of EA associated with cryptorchidism is relevant in clinical practice to prevent accidents during orchidopexy and to counsel patients and predict infertility in the future, such as in cases of epididymis atresia, disjunction of epididymal head and total disjunction between the testis and epididymis (16, 17).

Turek et al. (12) found high incidence of EAs associated with cryptorchidism in the literature (1, 18, 19). The author considered it to be a consequence of the lack of definition of the normal pattern of the anatomy of the epididymis in the several studies. In a recent paper (11), with the same standard proposed by Turek et al. (12) to analyze the relationship between the testis and epididymis, the authors found incidence of more than 30% EAs (disjunction and/or atresia) in patients with cryptorchidism, confirming the high incidence of this anomaly.

EAs can be classified as disjunction anomalies, epididymal atresia and elongated epididymis (12, 13, 18). In this study, we used the same standard classification proposed by Turek et al. (12) to analyze the relationship between the testis and epididymis and found incidence greater than 18% of the total disjunctions between testis and epididymis and head disjunction of epididymis in patients with cryptorchidism, confirming the high incidence of EAs associated with infertility in this condition. We did not observe epididymal atresia in our sample, which suggests the rarity of this anomaly (1).

The epididymis is anatomically connected to the gubernaculum, which in turn is attached to the testis and scrotum. An interesting and controversial theory suggests that the epididymis is one of the organs responsible for testicular migration, through its peristaltic and secretory activity in the second gestational trimester (20). This theory would explain some cases of cryptorchidism, where the epididymis is separated and located lower than the testis (21).

Mollaeian et al. (1) studied 652 undescended testes and observed epididymal disjunction in 16.87% of the cases. Caterino et al. (22), in a study of 895 patients, and Kim et al. (2) in a study of 110 patients, reported EAs, respectively, in 96% and 65% - a very high rate. Probably this high rate was associated with a classification considering normal relationships between testis and epididymis as anomalies. In this study, we observed EAs in 39 patients (43.7%). Disjunction of epididymal tail was observed in 23 patients (25.3%), and disjunction of epididymal head and total disjunction between the testis and the epididymis (anomalies that could be associated with infertility) were observed in 16 patients (18.4%), a significant number. Infertility can only be determined after a couple is unsuccessful in achieving pregnancy by natural conception after 12 months of unprotected intercourse. Patients with solitary testicles can have normal fertility. A very interesting data in our sample is the presence of total disjunction between the testis and the epididymis (Type 5 epididymal anomaly) in 2 of the 13 patients with bilateral cryptorchidism of our sample (15.38), which would lead these patients to have a great chance of having infertility.

Some studies have found an association of patency of the PV with EAs and cryptorchidism, suggesting there is a common stimulus, probably androgenic, participating in the epididymal development, testicular migration and closure of the PV (3, 23). The PV is usually obliterated after the end of testicular migration (24, 25). The rate of patency of the PV in patients with cryptorchidism ranges from 21.3 to 81.3% (23). We observed patency of the PV found in 74 cases (64.7%) of cryptorchidism, but with no significant correlations with the incidence of EAs. This finding is discordant with several studies in the literature. It can be attributed to the type of classification used to determine EAs. We used the classification that is currently accepted in the literature (12, 13). The anomalies associated with spermatic obstruction (Types IV and V) were observed in 6 cases (16.7%) of obliterated PV and 12 cases (16.2%) of patency of the PV, with no significant difference.

The main limitations of our study are: a) small sample size; b) uneven age distribution; c) impossibility of histological analysis to observe patency of epididymal ducts (due to ethical reasons); and d) we do not have long-term follow-up of patients with types 4 and 5 epididymal abnormalities to ascertain if they have developed infertility. A perfect study would be to, prospectively, follow patients diagnosed with undescended testis until they reach adulthood.

## CONCLUSIONS

We found epididymal anomalies associated with spermatic obstruction in almost 20% of undescended testes and their incidence had no correlation with age, testicular position and patency of the PV. This information needs to be correlated to the infertility risk in this congenital anomaly.
